# Information Indices with High Discriminative Power for Graphs

**DOI:** 10.1371/journal.pone.0031214

**Published:** 2012-02-29

**Authors:** Matthias Dehmer, Martin Grabner, Kurt Varmuza

**Affiliations:** 1 Institute for Bioinformatics and Translational Research, UMIT, Hall in Tyrol, Austria; 2 Laboratory for Chemometrics, Institute of Chemical Engineering, Vienna University of Vienna, Vienna, Austria; Wayne State University, United States of America

## Abstract

In this paper, we evaluate the uniqueness of several information-theoretic measures for graphs based on so-called information functionals and compare the results with other information indices and non-information-theoretic measures such as the well-known Balaban 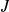 index. We show that, by employing an information functional based on degree-degree associations, the resulting information index outperforms the Balaban 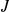 index tremendously. These results have been obtained by using nearly 12 million exhaustively generated, non-isomorphic and unweighted graphs. Also, we obtain deeper insights on these and other topological descriptors when exploring their uniqueness by using exhaustively generated sets of alkane trees representing connected and acyclic graphs in which the degree of a vertex is at most four.

## Introduction

To quantify the topology of networks, numerous topological descriptors, which are also often referred to as graph measures or indices, have been developed [1]–[7]. A property thereof called the *uniqueness*, *discriminative power* or *degeneracy* has been investigated extensively in mathematical chemistry and structure-oriented drug design in the context of characterizing the structure of molecules quantitatively. In general, a descriptor is called *degenerate* if it possesses the same value for more than one graph. In this paper our main task is to examine the extent to which topological indices are degenerate.

We briefly review the most important contributions to tackle this problem, and start with a classical contribution due to Bonchev et al. [8], [9]. They proposed the so-called magnitude-based information indices for improving the discriminative power of other classical descriptors for alkane trees [8] and isomers [9]. Alkane trees are connected and acyclic graphs in which the degree of a vertex is at most four [10]. Following this, Raychaudhri et al. [11] analyzed the discriminative power of information-theoretic measures based on distances for chemical graphs containing one ring. Konstantinova et al. [12] explored the uniqueness of various information-theoretic and non-information-theoretic measures by using polycyclic structures representing cata-condensed benzenoid hydrocarbons. As a result, the Balaban 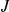 index (see equation 20), the sum of local vertex entropies due to Konstantinova [12], [13] and the magnitude-based information indices turned out to be unique for this class of graphs; see [12]. However, note that the sizes of the corresponding sets 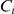, denoted by 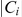, were rather small, 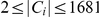. Diudea et al. [14] recently explored a novel super-index based on shell matrices and polynomials. By applying this index to the heterogeneous graph database MS2265 [15] containing 2265 non-isomorphic skeleton graphs, inferred from chemical compounds, and to chemical isomers, it turned out that this index does not have any degeneracy [14]. Other results obtained when applying further topological descriptors to chemical graph databases can be also found in [14]. Hu and Xu [16] applied an index using layer matrices and powers of extended adjacency matrices to over two million weighted alkane isomers. The index was unique for all graph classes used [16], but we point out that the developed index is based on using bond types and 3D information.

In order to underpin the practical importance of exploring uniqueness, it seems reasonable that an appropriate graph measure to characterize the structure of networks quantitatively should be able to discriminate graphs properly (e.g., when slightly changing the structure of a network). Note that this problem has already been discussed in the context of complex networks; see [17]. As to applications thereof, Dehmer et al. [15] have already outlined that unique measures can serve as candidates for calculating the identification codes of networks (e.g., chemical structures), which could be used to perform fast structure searches in large databases. Also, such highly discriminating measures representing graph invariants (the measured value is invariant under graph isomorphisms [10]) can be useful to tackle the graph isomorphism problem, because, if the values of two graphs with the same number of vertices are different, they must be non-isomorphic. Hence, such indices could be employed to tackle the graph isomorphism problem in large databases, as the computational complexity of the measures is polynomial. That means instead of performing a thorough isomorphism test which may be computationally costly, highly unique graph measures could be used to filter out non-isomorphic graphs. Note that the time complexity of some of these measures has already been discussed in [15].

The main contribution of this paper is to evaluate the discriminative power of selected topological indices in the context of complex networks, i.e., graphs that are neither regular nor random [18]. We applied several information-theoretic and non-information-theoretic measures, such as the Balaban 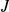 index [19], to nearly 12 million exhaustively generated, non-isomorphic and unweighted graphs with the same number of vertices (see ‘Numerical results and interpretation’). Importantly, we only use unweighted graphs in this study, as it poses an extra challenge to the underlying descriptors to discriminate such graphs on a large scale. We emphasize that the Balaban 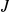 index has often been referred to as one of the most discriminative indices (see e.g. [20]), as it is powerful when applied to several classes of isomers and alkane trees. Our study highlights the limitations of the Balaban 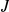 index and other topological descriptors in terms of their ability to discriminate non-isomorphic graphs uniquely.

We prove that one of the information indices due to Dehmer et al. [15], [21], which uses the information functional 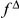 based on degree-degree associations, outperforms the Balaban 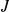 index tremendously when these measures are applied to exhaustively generated graphs. We also employ other information measures for graphs using so-called information functionals that have been developed by Dehmer et al. [15], [21]. The discriminative power of some of these information measures and classical ones has already been evaluated in [22] specifically for chemical graphs possessing structural constraints. By contrast, we perform a large-scale study to compare the discriminative power of these information measures by employing three information functionals (see equations 7, 8, and 18) and non-information-theoretic indices such as the Balaban 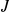 index using exhaustively generated graphs without structural constraints. The discriminative power by employing these particular information functionals and Balaban 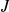 index has not yet been investigated on a large scale.

The results can be interpreted as an attempt to evaluate the uniqueness of quantitative graph measures in the context of complex networks. To the best of our knowledge, very little work has so far been done to tackle this problem. One exception is the work of Kim et al. [17], who evaluated the discriminative power of graph complexity measures that were developed in the context of network physics. As a result, most of the complexity measures proposed in [17] turned out to show little discriminative power.

This paper is organized as follows. In the section ‘Topological descriptors’ we briefly recall the definitions of the information-theoretic measures due to Dehmer et al. and the other graph measures that we are going to use. The ‘Data and software’ section describes the datasets and sketches the steps to calculate the topological descriptors. In ‘Numerical results and Interpretation’, we present and interpret the numerical results when evaluating the discriminative power of the measures. This includes a statistical analysis to investigate the dependence of the uniqueness of the Balaban 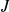 index and 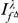 on the sample size by using exhaustively generated graphs with 10 vertices. The paper finishes with a ‘Summary and conclusion’.

## Methods

### Topological Descriptors

In this section, we briefly recall the definition of the information measures [4], [15], [21] that we are going to use in this study. Further, we outline the concept of distance-based descriptors, including the well-known Balaban 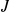 index. In summary, Table 1 gives an overview of the descriptors that we use.

**Table 1:** The topological indices used and their symbols.

Index Name	Symbol
Balaban index [19]	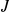
Balaban-like 1 [36]	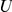
Balaban-like 2 [36]	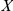
Bertz index [46]	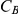
Magnitude-based Entropy [8]	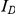
Magnitude-based Entropy [8]	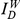
Compactness [7]	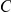
Complexity Index [2]	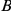
Vertex Complexity [11]	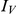
Harary index [7]	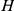
Hyper Distance Path index [7]	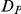
Sum of Vertex Entropies [13]	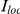
Normalized Edge Complexity [2]	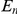
Prod. of Row Sums [7]	PRS
Radial Centric index [1]	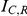
Top. Information Content [31]	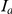
Index of total adjacency [2]	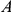
Degree Information index [1]	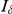
Zagreb 1 [7]	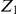
Zagreb 2 [7]	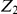
Information index using 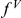 [15], [21]	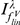
Information index using 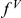 [15], [21]	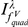
Information index using 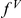 [15], [21]	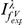
Information index using 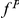 [15], [21]	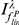
Information index using 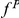 [15], [21]	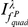
Information index using 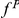 [15], [21]	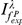
Information index using 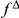 [21], [34]	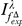

#### Information Indices

To start, we point out that, besides empirical properties of information measures for graphs [1], [4], [15], [21] (such as determining correlations between the measures [1]), mathematical problems (such as proving various upper and lower bounds of the measures) have also been explored; see [23], [24]. Note that the correlation ability between two graph measures generally relates to the problem of whether they capture structural information similarly [1], [9]. The so-called implicit information inequalities have been investigated extensively in [21], [25], [26]. Also, the class of graph entropy measures obtained by using certain information functionals based on the metric properties of graphs (such as the neighborhoods of atoms) has been used to solve problems in quantitative structure–activity relationships (QSARs) and quantitative structure–property relationships (QSPRs) [27]. In particular, Dehmer et al. [28] classified the mutagenicity of molecules by using these measures and employing supervised learning techniques.

Let 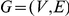 be an arbitrary, finite, and unweighted graph; 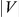 denotes the number of vertices and 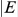 the number of edges, respectively. Throughout this paper, we use the symbol 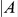 to express the cardinality (also called the size) of a set 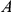. We denote by 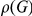 the diameter of 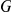; see [29]. The abstract information functionals [21] 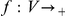 play a critical role when defining information measures on graphs. Based on these functionals, vertex probabilities [21]

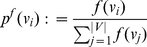
(1)have been assigned to each particular vertex of 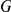. This makes the resulting measure independent of determining partitions of graph invariants [1], [8], [30], [31], which might be computationally difficult to obtain. By definition,
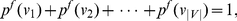
(2)and 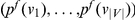 therefore forms a probability distribution. Using this approach and recalling Shannon's entropy [32] defined by
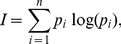
(3)the families of information measures
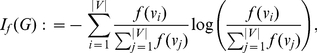
(4)

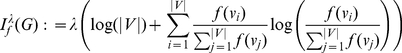
(5)have been developed [4], [15], [21]. These measures are families of entropic measures representing the structural information content of 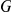. Here 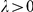 is a scaling constant, 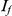 is the mean entropy of 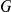, and 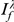 its information distance between maximum entropy and 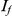.

In our analysis, we define three distinct functionals 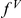, 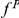, and 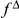, and the relative information measures 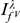, 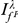, and 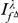 [4], [5], [21]. To define 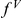, we first define the 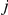-sphere of a vertex 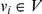 by [21]

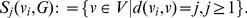
(6)

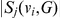
 are just the 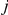-sphere cardinalities. In general, 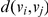 is the shortest distance between the vertices 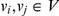; see [33]. Then,
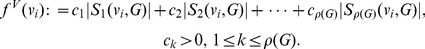
(7)To define 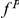, the pathlengths for 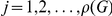 of the local information graph 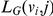 starting from a particular vertex have been used; see [21] for its detailed definition. For example, 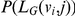 is the sum of all pathlengths starting from 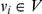 by inducing shortest paths for 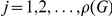. We obtain
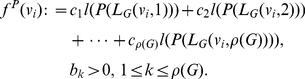
(8)Finally, we define 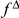 (see [34]), let 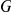 be an undirected and unweighted graph, and set 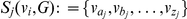, 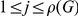, 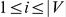. For 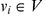, we define the sets of shortest paths [34]

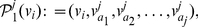
(9)

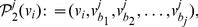
(10)

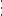


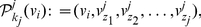
(11)and the corresponding degree sequences [34]

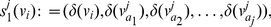
(12)

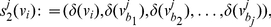
(13)

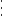


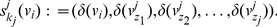
(14)The quantities [34]

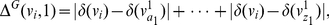
(15)

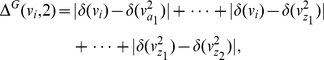
(16)

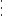


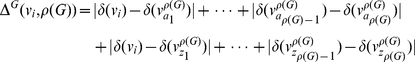
(17)have been used to define the information functional 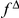; see equation 18. As we employ the differences 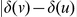, the resulting graph entropies 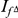 and 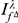 have been called *degree–degree association indices*; see [34]. Now, 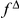 has been defined by [34]

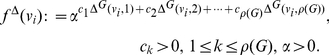
(18)We see that 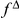 is well defined for any 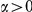. Since 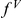, 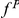 and 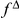 as well as the resulting entropies are parametric, we need to choose the coefficients 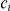 for weighting the structural differences or characteristics of a graph. Note that the 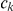 must be chosen such that at least two coefficients 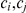 are distinct. This includes the parameter settings, e.g.,
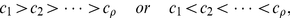
(19)which have already been used in [15]. Other configurations of the 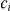 have also been investigated to determine the structural complexity of chemical structures meaningfully [15].

#### Distance-Based Topological Descriptors

Numerous topological descriptors have been explored by employing distances in a graph [7], [19], [29]. Seminal work was done by Skorobogatov and Dobrynin [29], who developed a theory on the metric properties of graphs. Also, several distance-based graph measures have been developed and analyzed where these indices have shown that distances in graphs capture significant information when applied in QSAR/QSPR; see [1], [7], [11], [19], [27].

We recall the definition of the Balaban 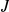 index [7], [19] in detail as we place emphasis on comparing its discriminative power with 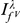, 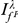, and 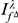 on a large scale by using exhaustively generated graphs. The names and symbols of the remaining descriptors used in this study can be found in Table 1. For their formal definitions, see [1], [2], [7], [27].

Now, we define the distance matrix [35] of a graph 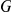 as 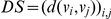. For each vertex 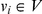, 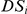 denotes the distance sum (row or column sum) obtained by adding the entries in the corresponding row or column of the distance matrix 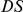. In addition, 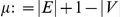 is the cyclomatic number [36]. Then, the Balaban 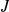 index is defined by [19]

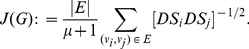
(20)


## Results

### Data and Software

Let us now state the definitions and generation procedure of the graphs for performing our analysis.

**Definition 1** 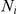 *is the set of all exhaustively generated non-isomorphic and connected graphs with* 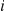 *vertices.*

Practically, these sets have been generated by using the program geng from the Nauty package [37]. In this study we use the classes 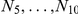 and obtain their cardinalities as follows: 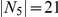, 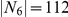, 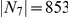, 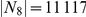, 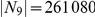, and 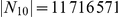. These numbers are in accordance with the results due to McKay [37], [38].

**Definition 2** 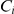 *is the set of all exhaustively generated non-isomorphic alkane trees graphs with* 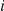 *vertices.*

The chemical structures represented by alkane trees with a carbon backbone have been generated with Molgen [39]. In particular, we generated the classes 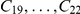; their cardinalities are 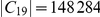, 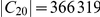, 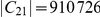, and 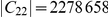.

Then for both classes (see Definitions 1 and 2), the structure information has been converted into the graphNEL format to calculate the descriptors in R [40] by employing the QuACN package [41]. This package contains R functions of over a hundred topological descriptors.

### Numerical Results and Interpretation

In this section, we present the numerical results when evaluating the discriminative power of the information indices, Balaban 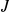 index and other topological descriptors. Results on exhaustively generated graphs are summarized in Tables 2 and 3, while those on alkane trees are given in Table 5. In total, we evaluated the discriminative power of 27 graph measures.

**Table 2:** 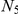
, 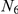 and 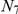 are exhaustive sets of non-isomorphic and connected graphs. 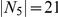, 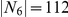 and 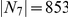.

	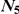		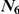		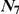	
Index	ndv	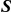	ndv	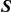	ndv	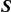
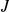	0	1,000000	10	0,910714	155	0,818288
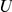	0	1,000000	10	0,910714	155	0,818288
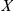	0	1,000000	10	0,910714	155	0,818288
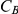	20	0,047619	111	0,008929	852	0,001172
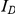	15	0,285714	100	0,107143	826	0,031653
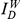	14	0,333333	94	0,160714	811	0,049238
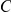	16	0,238095	108	0,035714	847	0,007034
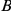	2	0,904762	34	0,696429	486	0,430246
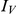	10	0,523810	91	0,187500	797	0,065651
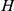	14	0,333333	100	0,107143	828	0,029308
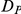	14	0,333333	101	0,098214	837	0,018757
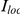	2	0,904762	34	0,696429	450	0,472450
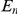	19	0,095238	110	0,017857	851	0,002345
PRS	2	0,904762	38	0,660714	486	0,430246
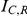	20	0,047619	111	0,008929	852	0,001172
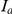	20	0,047619	111	0,008929	852	0,001172
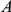	19	0,095238	110	0,017857	851	0,002345
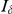	20	0,047619	111	0,008929	852	0,001172
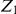	19	0,095238	110	0,017857	851	0,002345
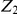	0	1,000000	37	0,669643	750	0,120750
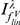	4	0,809524	37	0,669643	485	0,431419
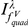	4	0,809524	37	0,669643	452	0,470106
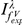	4	0,809524	37	0,669643	454	0,467761
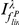	9	0,571429	38	0,660714	312	0,634232
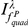	2	0,904762	23	0,794643	97	0,886284
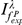	2	0,904762	5	0,955357	7	0,991794
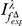	6	0,714286	16	0,857143	34	0,960141

**Table 3:** Exhaustive sets of non-isomorphic graphs. 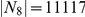, 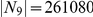, 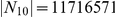.

	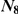		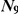		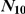	
Index	ndv	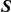	ndv	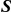	ndv	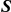
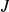	4262	0,616623	156674	0,399900	9307263	0,205633
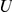	4093	0,631825	148132	0,432618	8812811	0,247834
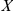	4093	0,631825	148132	0,432618	8812810	0,247834
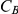	11116	0,000090	261079	0,000004	11716570	0,000000
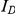	11070	0,004228	260971	0,000417	11716339	0,000020
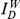	11014	0,009265	260803	0,001061	11715858	0,000061
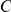	11110	0,000630	261072	0,000031	11716564	0,000001
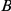	8384	0,245840	237199	0,091470	11472695	0,020815
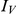	10958	0,014302	260650	0,001647	11715029	0,000132
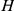	11076	0,003688	261018	0,000237	11716455	0,000010
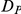	11100	0,001529	261054	0,000100	11716541	0,000003
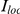	8305	0,252946	235233	0,099000	11395248	0,027425
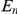	11115	0,000180	261078	0,000008	11716569	0,000000
PRS	9376	0,156607	252262	0,033775	11672850	0,003732
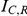	11116	0,000090	261079	0,000004	11716570	0,000000
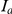	11116	0,000090	261079	0,000004	11716570	0,000000
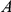	11115	0,000180	261078	0,000008	11716569	0,000000
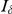	11116	0,000090	261079	0,000004	11716570	0,000000
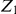	11115	0,000180	261078	0,000008	11716569	0,000000
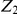	10996	0,010884	260931	0,000571	11716379	0,000016
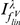	9165	0,175587	249439	0,044588	11640381	0,006503
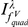	8300	0,253396	235044	0,099724	11385762	0,028234
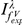	8300	0,253396	235055	0,099682	11385730	0,028237
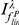	4989	0,551228	158391	0,393324	9479777	0,190909
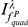	1699	0,847171	58196	0,777095	4243499	0,637821
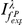	478	0,957003	27017	0,896518	2619898	0,776394
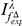	385	0,965368	6016	0,976957	609204	0,948005

#### Evaluation of the Discriminative Power Using Exhaustively Generated Graphs

To interpret the numerical results, we start by considering Table 3 and observe that the sensitivity values due to Konstantinova [12], 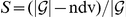, for Balaban 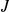 decreases with increasing number of vertices; see also the ‘Statistical analysis’ section. Throughout this paper, ndv (non-distinguishable values) stands for the number of non-isomorphic graphs whose values cannot be distinguished by a particular index [12]. For example, by considering the class 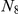, 61.6623% of the graphs could be distinguished (i.e., have unique values) by the Balaban 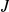 index. For 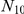, only 20.5633% out of almost 12 million exhaustively generated non-isomorphic graphs could be distinguished by 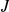. But we can see in Table 3 that the information indices using the information functional approach [4], [15], [21] sketched in the ‘Information indices’ section can discriminate our graphs comparatively well. In particular, 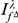, with an exponential weighting scheme
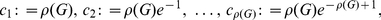
(21)denoted by 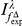, discriminates 94.8005% out of almost 12 million exhaustively generated graphs successfully. In view of the large number and complexity of the graphs (see 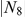, 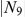 and 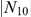), the uniqueness of 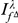 is striking. Observe that, for all weighting schemes [15], i.e., lin, quad, and exp, 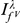 is much less discriminative. We realize that the underlying information functional 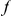 is crucial for reaching uniqueness of the information index. Also, we can clearly see that the uniqueness of other indices shown in Table 3 is quite low. We see that the Balaban 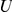 and 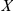 indices are among the best out of the set of known measures that we have chosen to perform this study.

**Figure 1:**
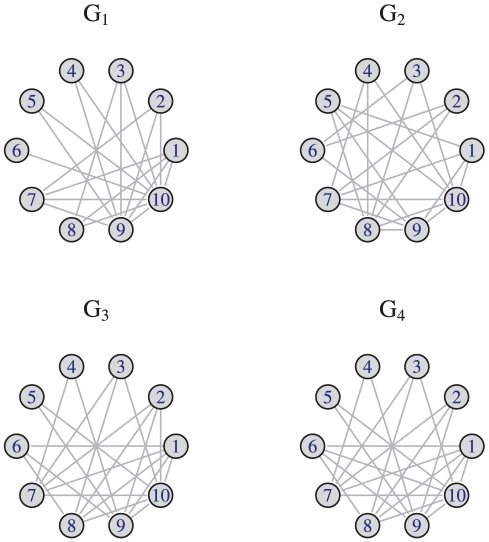
Four example graphs 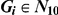
 **.**

Interestingly, the situation is somewhat the opposite when considering Table 2. Namely, for 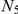 and 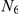, the discriminative power of the Balaban 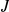 index is higher than by using some of the information measures based on the information functional approach (e.g., 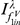 and 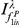). Also, we see that the underlying weighting scheme for the coefficients matters a lot, because 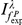 has a higher discriminative power than the Balaban 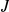 index for 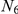 and 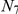. In summary, we hypothesize that the Balaban 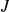 index performs well if the cardinality of the underlying graph set and the order of the involved graphs is rather small. By using a statistical approach, we will verify this hypothesis in the ‘Statistical analysis’ section. Let us give another example to shed light on the degeneracy of the measures when applying them to graphs 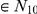, see Figure 1 and Table 4. Figure 1 shows four sample graphs 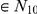 where 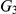 and 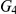 are structurally quite similar in the following sense. If we remove the edge 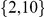 in 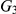 and the edge 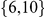 in 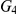, the resulting graphs are isomorphic. From Table 4, we see that these graphs can only be fully distinguished by the degree-degree association index. Evaluating the Balaban 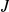 index on these graphs gives two degenerate graphs namely 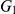 and 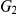. In contrast to this, 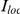 due to Konstantinova can not discriminate 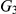 and 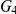. Finally, we observe that 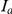 can not discriminate any of the four example graphs. This implies that every measure captures structural information differently and, hence, its discriminative power can differ dramatically because of

- the underlying paradigm to define a graph measure, e.g., information-theoretic vs. non-information-theoretic indices or partition-based vs. non-partition-based
- the underlying graph invariant to define a measure, e.g., degrees or distances or several graph invariants etc.

**Table 4:** Index values for the four example graphs depicted in Figure 1.

	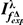	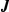	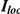	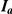
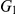	0.0002695	2.639475	31.16882	3.121928
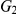	0.8801102	2.633647	30.90633	3.321928
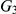	0.2076738	2.564776	30.92375	3.321928
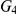	0.0017872	2.564776	30.92375	3.321928

A comparison of the measures with others (e.g., see Table 3) is critical, as the measures rely on different concepts (e.g., information-theoretic vs.non-information-theoretic indices). In the following, we give plausible reasons why the measures using the information functional approach often capture structural information of exhaustively generated graphs more uniquely and significantly than other information measures for graphs that are based on determining partitions of graph invariants. This can also be underpinned by the numerical results; see Tables 2 and 3. Examples of the latter measures are the magnitude-based information indices 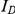 and 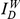 due to Bonchev et al. [8], the degree information index 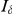 [1] and the topological information content of a graph 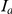 [31], [42].

To construct classical partition-based measures of a graph 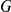, we start with a graph invariant 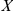 and induce a partitioning according to an equivalence criterion. This results in the equivalence classes 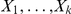 being obtained. The mean entropy is then given by
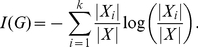
(22)The process of inducing the partitionings might be the reason for obtaining non-unique indices, as many structurally different graphs could possess the same or similar partitionings when using a certain equivalence criterion, e.g., vertex degree equality [1] or topologically equivalent vertices [31], [42].

In order to derive information measures using the information functional approach, we assign a probability value (see equation 1) to each individual vertex in a graph by using a certain information functional 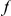 capturing its structural information. Examples thereof are equations 7 and 18. That means the information measures given by equations 4 and 5 can be understood as a cumulation of local quantities representing the vertex probabilities. Clearly, each such quantity captures a certain percentage rate of the structure of 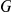. As the numerical results show, these measures conserve structural information more properly than the partition-based ones and result in highly discriminating measures for several graph classes. Note that other classical descriptors (see Tables 2 and 3), such as the Harary index, Randi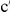 index [43], [44] and the complexity index 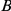 etc., rely on the simple derivation of structural quantities (e.g., distances or degrees) to obtain a single numerical value characterizing the complexity the graph. Consequently, their discriminative power is very low; see Tables 2 and 3.

**Figure 2:**
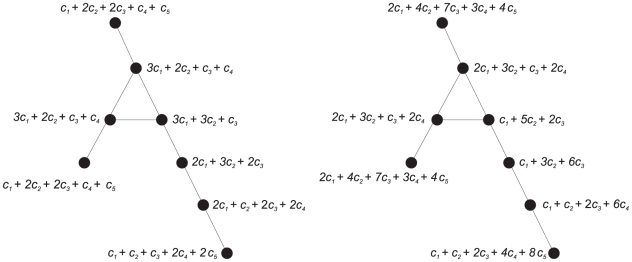
Left: A cyclic graph and its values of 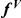
 **for each vertex. Right: Values of** 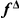 **for each vertex for the same graph.**

When evaluating the uniqueness (see ndv or 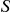 values) of 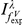 and 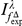 (see Table 3), we observe that the difference between the resulting values is tremendous. Note that the graphs of 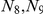, and 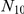 contain cycles. A plausible reason for this is given in Figure 2.

We see on the left-hand side that the 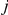-sphere cardinalities are rather small if 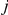 goes to 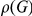 and, hence, their contribution to the value of the particular functional for 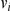 is small too. Also, there is not much variation between the 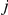-sphere cardinalities. This could be a reason that the resulting probability values
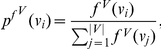
are quite similar to each other and, thus, this has a direct influence on the resulting value of the information index and on its uniqueness. In contrast, the right-hand side of Figure 2 shows that the values of 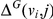 are more diverse and, in particular, those values when 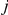 goes to 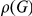 are larger than the 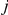-sphere cardinalities. This might be a plausible reason why the corresponding vertex probability values are more different and, hence, the resulting entropies as well. As Tables 2 and 3 show, we again emphasize that the discriminative power of an index clearly depends on the underlying graph class.

#### Evaluation of the Discriminative Power by Using Chemical Graphs

Here we evaluate the uniqueness of the Balaban 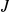 index, the information measures using the information functional approach, and the remaining topological descriptors shown in Table 1 by also using chemical graphs. Table 5 depicts the numerical results when applying the measures to chemical alkane trees representing the skeletal graphs. The number of vertices ranges from 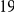 to 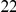. We see again that the discriminative power of the Balaban 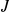 index decreases when the number of graphs and vertices increase. The Balaban-like indices possess high discriminative power for all four graph classes. Also, we observe that the sum of the local vertex entropies (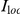) due to Konstantinova [13], [45] has high uniqueness. Interestingly, it is as good as 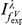 and 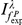. It can be easily shown that, for trees, the information indices using 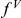 and 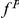 have equal discriminative power. In particular, 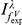, 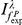 and the just mentioned indices clearly outperform the Balaban 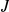 index by using the chemical alkane trees.

**Table 5:** Chemical alkane trees 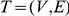 with 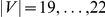. 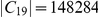, 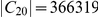, 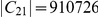, 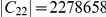.

	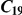		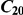		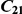		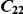	
Index	ndv	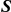	ndv	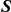	ndv	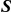	ndv	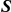
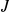	5967	0,959760	44800	0,877702	45703	0,949817	306911	0,865311
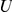	0	1,000000	12	0,999967	4	0,999996	82	0,999964
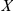	0	1,000000	12	0,999967	4	0,999996	82	0,999964
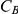	148278	0,000040	366312	0,000019	910718	0,000009	2278645	0,000006
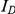	68030	0,541218	171655	0,531406	452442	0,503207	1140578	0,499452
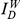	39731	0,732061	97815	0,732979	277238	0,695586	702776	0,691583
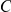	148267	0,000115	366289	0,000082	910713	0,000014	2278626	0,000014
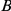	5959	0,959814	44752	0,877833	45667	0,949857	306469	0,865505
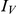	104790	0,293316	279826	0,236114	730474	0,197921	1942075	0,147711
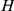	125290	0,155067	319121	0,128844	813614	0,106631	2081153	0,086676
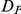	147946	0,002279	365914	0,001106	910290	0,000479	2278165	0,000216
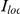	0	1,000000	12	0,999967	4	0,999996	84	0,999963
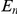	148283	0,000007	366318	0,000003	910725	0,000001	2278657	0,000000
PRS	5967	0,959760	44810	0,877675	45701	0,949819	306953	0,865292
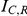	148283	0,000007	366318	0,000003	910725	0,000001	2278656	0,000001
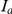	148278	0,000040	366312	0,000019	910718	0,000009	2278645	0,000006
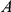	148283	0,000007	366318	0,000003	910725	0,000001	2278657	0,000000
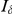	148283	0,000007	366318	0,000003	910725	0,000001	2278657	0,000000
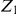	148283	0,000007	366318	0,000003	910725	0,000001	2278657	0,000000
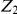	148282	0,000013	366317	0,000005	910724	0,000002	2278656	0,000001
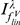	5006	0,966241	37820	0,896757	39210	0,956946	263231	0,884480
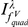	42	0,999717	268	0,999268	324	0,999644	1752	0,999231
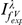	0	1,000000	12	0,999967	4	0,999996	84	0,999963
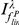	5006	0,966241	37820	0,896757	39210	0,956946	263231	0,884480
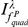	42	0,999717	268	0,999268	324	0,999644	1752	0,999231
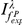	0	1,000000	12	0,999967	4	0,999996	84	0,999963
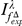	67176	0,546977	196124	0,464609	544432	0,402200	39396	0,982711

Finally, the numerical results show again that the discriminative power of a structural index strongly depends on the underlying graph class. See, for instance, the results when comparing the uniqueness of 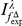 for the alkane trees and exhaustively generated graphs (see Table 3).

#### Descriptive Statistical Analysis

**Figure 3:**
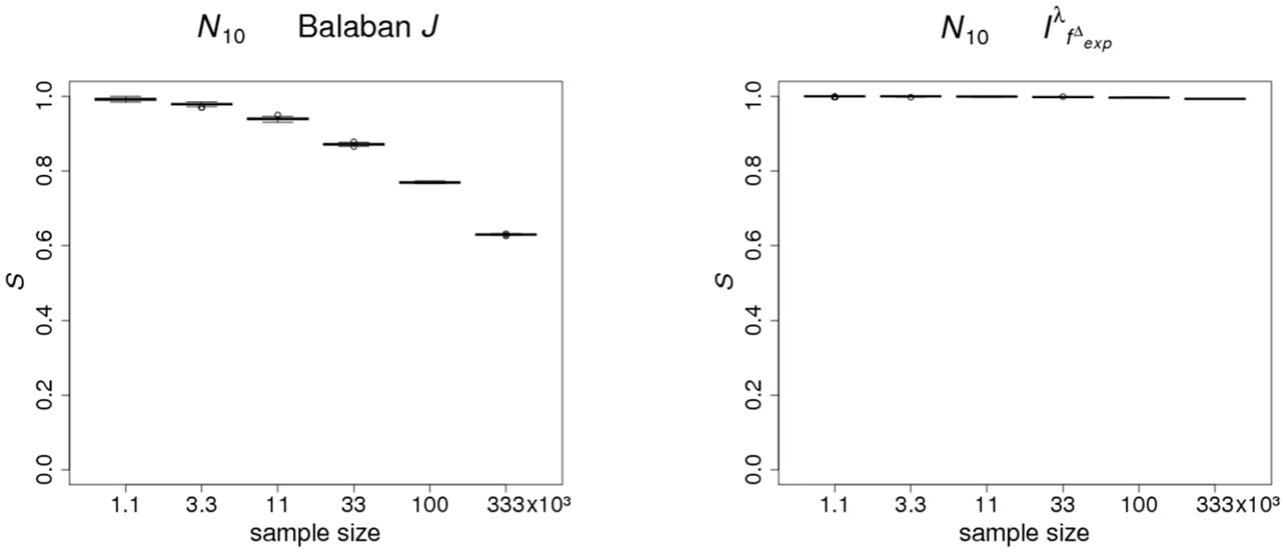
Boxplots to investigate the dependency of the uniqueness of Balaban 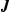
 **and** 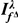 **from the sample size by using exhaustively generated graphs with ten vertices.**

In order to provide further evidence for stability of the uniqueness of 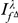 by using exhaustively generated graphs, we perform a statistical analysis by using boxplots. The graph class to perform the study is 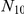. It is clear that, for computational reasons, the statistical analysis cannot be performed by using the entire set 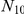. Hence, we choose subsets of 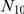 whose sizes are called sample sizes. Also, we perform the boxplot analysis for Balaban 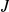 as well, and present the resulting plots to investigate the dependence between uniqueness and sample size; see Figure 3. Concretely, 100 samples of 1100, 3300, 11 000, 33 000, 100 000, and 333 000 randomly chosen graphs out of 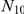 have been analyzed by standard R boxplot routines. That means the medians have been calculated and plotted, with the first and third quantiles as hinges. The whiskers represent the calculated borders of the 95% confidence interval.

As we can see in Figure 3 the uniqueness values are not dispersed for a given sample size, but they depend on the sample size. Further, we observe that the uniqueness of the Balaban 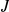 index is not stable when the sample size is varied. In general, we call a measure 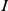 unstable if there is a strong dependency between the uniqueness of 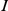 and the sample size to perform the statistical analysis. In contrast, 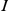 is stable if there is only a very little dependency between the uniqueness of 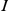 and the sample size.

We see from the boxplot that the uniqueness decreases if the sample size increases. Based on our intuition, it seems reasonable that, the smaller the sample size, the better is the discriminative power of the measure under consideration. Thus 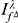 possesses a non-trivial property, namely a very high discriminative power for exhaustively generated graphs that is almost independent of sample size. By using the above stated definition, we see that 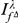 is stable on 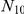 as the uniqueness is constantly high and does not depend much on the sample size. We see from Table 3 that 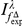 is the only topological descriptor possessing this property. Other topological measures, and particularly the Balaban 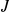 index, have the trivializing property that, for exhaustively generated graphs, the uniqueness is only reasonable for small sets of graphs.

Hence some of the entropy measures using the information functional approach could be applied successfully for discriminating sets of large complex networks as well. Keep in mind that in fact such classes of exhaustively generated complex networks possess huge cardinalities. Note that the cardinality of the exhaustively generated non-isomorphic graphs with 10 vertices is already greater than 11 million. As we conclude from this statistical analysis, 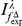 possesses the stability property that is necessary to achieve feasible results when applied to sets of large complex networks.

### Summary and Conclusion

In this paper, we have dealt with the problem of evaluating the discriminative power of topological graph measures by using exhaustively generated, non-isomorphic graphs without vertex and edge weights. We have made an attempt to translate topological indices into the field of complex networks when evaluating their uniqueness. We found that one of the information measures for graphs using the information functional based on degree–degree associations outperformed the Balaban 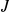 index tremendously. Also, by using the graph class 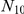, we found that the uniqueness of the Balaban 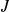 index is quite sensitive to varying sample size when performing the statistical analysis; see ‘Statistical analysis’ section. In particular, the uniqueness of the Balaban 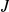 index deteriorated when increasing the sample size. This makes Balaban 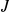 in particular non-feasible for discriminating complex networks structurally as they are multicyclic, do not have structural constraints, and the cardinality of an underlying set of such networks is huge. This property was also observed by using other topological indices shown in Table 1. The numerical results when using exhaustively generated graphs and alkane trees can be found in Tables 2, 3, and 5.

Altogether, this study clearly shows the limitations of topological indices and restrictions when applying them on a large scale. A topological index can be unique for a particular graph class but it fails when applying the measure to another class. In this sense, it is far from trivial that we obtained an index (see the definition of 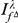) that turned out to be highly discriminating for exhaustively generated graph classes. Note that the underlying graphs do not possess structural constraints.

As to future work, we will evaluate further topological indices on a large scale to obtain deeper theoretical insights. From such an analysis, one can also learn how the measures capture structural information. This relates to better understanding of their structural interpretation. We are convinced that these developments could also trigger future developments positively when developing and investigating topological graph measures in the context of complex networks.
